# A Global Overview of Missed Nursing Care During Care of In-Patients with Cancer: A Scoping Review

**DOI:** 10.3390/nursrep15120413

**Published:** 2025-11-24

**Authors:** Joshua Kanaabi Muliira, Eilean Rathinasamy Lazarus, Prossy Nandawula

**Affiliations:** 1 Department of Adult Health and Critical Care, College of Nursing, Sultan Qaboos University, Al Khoudh, Muscat 123, Oman; eilean@squ.edu.om; 2 School of Health Sciences, Soroti University, Arapai, Soroti P.O. Box 211, Uganda; pnandawula@sun.ac.ug

**Keywords:** oncology nursing, nursing, missed care, unfinished nursing care, patient safety

## Abstract

**Background/Objective:** This review explored the literature on Missed Nursing Care (MNC) in inpatient oncology settings to gain insights on how to enhance the quality of nursing care for hospitalized patients with cancer and survivors. The aim was to identify the common MNC and the factors associated with MNC in inpatient oncology units. **Methods:** A scoping review approach was used, in which a five-stage methodological framework informed the process. Five databases were searched for relevant studies (EMBASE, Medline, SCOPUS, CINAHL, and PsycINFO) published from January 2013 to June 2025. Other search methods were conducted using Google Scholar, Trove, and ProQuest Dissertations for records focusing on the topic. The review included qualitative and quantitative articles. Thomas and Harden’s three-step method for thematic synthesis was followed to summarize data into themes. **Results:** Fifteen studies were selected and included in the scoping review. Three themes were generated: the commonly MNC; reasons for MNC; and factors associated with MNC. The common categories of MNC were related to basic patient care, documentation, and communication with patients or family members. The common factors associated with MNC were job satisfaction, patient load, and staffing adequacy. **Conclusions:** MNC is common in inpatient oncology settings and presents a key challenge to the safety of cancer patients and their health outcomes. Efforts to curtail MNC, such as integration of evidence-based policies, clinical guidelines, and standards in oncology nursing care, are needed. Interventional studies are needed to provide insight into effective remedies to the factors that fuel MNC, such as staffing, work overload, communication, work environment, and nurses’ skills. Studies from pediatric oncology settings, Africa, and other resource-limited settings where the future global burden of cancer will be highest are also needed.

## 1. Introduction

Missed nursing care (MNC) is any aspect of the required patient care that is omitted in part or whole, or delayed [1]. MNC can be clinical, emotional support, and/or administrative-related patient care activities that are partially performed, not performed, or not performed at the recommended time, and these severely impact the quality of nursing care and patient safety [1]. MNC can also be interpreted as an error since it can be an act of omission [2] or negligence [3].

The phenomenon of MNC is critical in oncology nursing because many patients with cancer and cancer survivors are immunocompromised due to the disease process or cancer therapies. MNC can worsen the complications or hasten the mortality of at-risk patients [3]. For instance, failure by a nurse to promptly communicate to the physician the results of severe neutropenia in a patient with cancer or a cancer survivor increases the risk of exposure to sources of infection, and subsequent sepsis and loss of life. Thus, some of the adverse events suffered by cancer patients and cancer survivors can stem from an error of commission (inappropriately performed action) or MNC, also called an error of omission or a necessary action not performed or not performed on time [4].

The impact of MNC can affect the healthcare organization, the patient, and the nurse [5]. Higher rates of MNC are associated with higher chances of low-quality care [6], low satisfaction with care, and predisposition to medication errors, infections, falls, pressure injuries, hospital re-admissions, and mortality [7]. MNC may lead nurses to experience low job satisfaction, high job turnover [8,9], absenteeism, and moral distress [5]. Moreover, the hospitals where MNC is frequent tend to be associated with low-quality nursing care, adverse events, decreased reputation in the community, high nurse turnover, and increased cost of care [5]. This implies that MNC is a major contributor to reduced patient safety, unsafe nursing care, and all of which compromise patients’ health outcomes.

Numerous factors affect oncology nursing practice globally, and some of these might be contributing to MNC. For instance, globally, oncology nurses face challenges such as burnout, frequent addition of newer innovations, limited access to specialized education, recruitment barriers (perception of oncology nursing as a demanding specialty with a complex and hazardous work environment), and others [10,11]. It is a huge challenge for nurses to deliver safe cancer care across the cancer continuum (diagnosis to survivorship) with only a general nursing education and experience due to the complex nature of cancer therapies, techniques, and technologies used to deliver care, health education, counseling, and others, which require specialized knowledge and skills [12]. The nursing shortage, inadequate numbers of oncology nursing faculty and oncology nursing programs, despite the increasing global cancer burden, all could be contributing to MNC and the quality of oncology nursing care [12].

Additionally, some oncology units sustain negative cultures such as negligence, which leads to MNC [13]. The literature shows that there are oncology units with practice environments that promote silent behavior towards patient safety, and this curtails error reporting and sustains MNC, as compared to open communication, which eliminates unsafe practices [14]. A recent study among oncology nurses working in two large Saudi Arabian tertiary care hospitals found low levels of patient safety culture, and this was associated with supervisor inaction, lack of support from hospital management, lack of open communication, and inexperience among nurses [15]. Thus, the lack of patient safety culture may also play a major role in the escalation of MNC in oncology nursing.

Despite the vulnerability of inpatients with cancer and cancer survivors, and the impact of MNC on their health outcomes, no studies have attempted to synthesize the findings of primary research on MNC in these specific patient populations and care settings. Our scoping review is addressing this gap in knowledge. On the other hand, multiple systematic review studies have addressed MNC during the COVID-19 pandemic [16] and MNC in other patient care settings such as intensive care units [17]. The lack of reviews, summarizing and synthesizing available literature limits our understanding of MNC in inpatient oncology settings. This scoping review synthesized the available literature from different countries on MNC in inpatient oncology units to identify the aspects of oncology nursing care that need to be targeted by interventions to ensure high-quality nursing care and patient outcomes. The review aimed to identify the common MNC and the factors associated with MNC in inpatient oncology units.

## 2. Methods

A scoping review is a type of knowledge synthesis that uses a systematic and iterative approach to identify and synthesize an existing body of literature on a given topic [18]. Scoping reviews help map existing literature and identify gaps on a given topic [18]. A scoping review is also a useful tool to provide a clear summary of the volume, availability, and focus of relevant studies [18]. Compared to other types of systematic reviews, a scoping review can cover a greater range of literature, including gray literature on a topic, such as dissertations or theses. In terms of gray literature, dissertations or theses were considered to ensure rich findings.

This scoping review used a five-stage framework [18,19] to investigate MNC and the associated factors within inpatient oncology units. The framework’s five stages include: (1) identify the research question to guide the search strategies; (2) identify the relevant studies, both published and unpublished; (3) develop eligibility criteria for study selection; (4) chart the data extracted from the records being reviewed; and (5) collate, summarize, and report the results as an overview of all materials reviewed [18,19].

### 2.1. Identifying the Research Question

The review team consisted of three senior nurses with experience in cancer nursing/oncology nursing, general nursing (clinicians, academicians, and researchers) and a senior librarian. All the authors engaged in discussing and formulating the research questions. The research questions were: What is the most common MNC by nurses caring for inpatients with cancer or recovering from cancer? What factors contribute to MNC among nurses caring for inpatients with cancer or recovering from cancer? The investigation focused exclusively on nurses who care for cancer patients, specifically oncology nurses working in hospital inpatient units.

### 2.2. Identifying Relevant Studies

The keywords, Medical Subject Headings (MeSH), thesaurus terms, and search phrases were selected based on the research questions (refer to Table 1). A senior librarian and three academic authors conducted systematic searches on five databases: CINAHL, MEDLINE, EMBASE, Scopus, and PsycINFO. Additional searches were conducted on Google Scholar, Trove, and ProQuest Dissertations to find relevant theses on the topic. The database searches included only articles published in English between January 2013 and June 2025 to ensure that the data were up to date. The earliest published article on MNC in an oncology context appeared in 2013, and this served as the starting point of this review.

Information about each search, along with additional methods used, can be found in the Supplementary File titled “search details”. Studies were included in the review if they met the following inclusion criteria: (1) reporting about MNC, reasons for MNC, and factors associated with MNC by nurses caring for patients with any type of cancer; (2) primary studies; (3) published between January 2013 and June 2025; and (4) the participants were nurses caring for patients hospitalized with cancer or recovering from cancer. Articles that discussed studies involving non-nurse personnel, as well as those focused on literature reviews or systematic reviews, or nurses caring for general patients without the diagnosis of cancer, were not included. In the current review, the reasons for MNC represent the rationale given by the nurses (direct reports by the nurse) as to why the care was missed or not provided. On the other hand, factors associated with MNC represent aspects such as demographic factors, nurses’ attitude, inexperience, and others that are identified through statistical and other analyses to be significantly associated with MNC (the factors associated with MNC are not direct reports by the nurses).

### 2.3. Selecting Studies

A total of 330 articles focusing on MNC or rationed care or unfinished care or care left undone were identified in the five databases. Initially, the first and second authors reviewed the titles and subject headings of 330 records. The initial review was conducted to eliminate book chapters, opinion articles, and others that were not based on primary studies (150 records). The subsequent study (article) selection process was thorough, followed the inclusion criteria stated above, and involved all the authors at all points. A total of 180 records were selected and exported into EndNote 20, and this helped to identify and eliminate seventy-two (72) duplicates from that set. Secondly, all the authors independently scrutinized the abstracts of the remaining 108 and used the inclusion and exclusion criteria to eliminate twenty-two records (focusing on other healthcare personnel or professionals, but not nurses).

**Figure 1:**
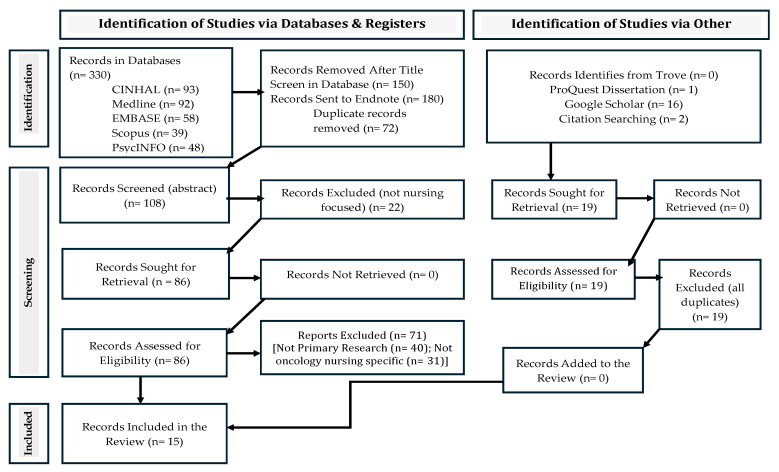
Flowchart referring to the study search and selection process.

All authors independently reviewed the remaining eighty-six (86) records in full text, then collaborated in pairs to verify each other’s decisions. In situations where there were divergent decisions, all authors met and discussed the different viewpoints to reach consensus on the inclusion or exclusion of a specific record/paper. This process led to the exclusion of another seventy-one (71) records (40 articles were not primary research, and 31 articles were not specific to inpatient oncology nursing care for cancer patients or cancer survivors). Figure 1 illustrates a detailed breakdown of the exclusions based on the specified criteria. The authors also identified nineteen (19) records using other search methods or by citation searching per the inclusion criteria. These records were all found to be duplicates. A total of fifteen studies (articles) were included in the final review (see Figure 1).

### 2.4. Charting the Data

The three authors performed data extraction and charting using a standardized protocol. Each article (study) was extracted by three different authors who later compared their data before the final data from each study was confirmed as complete. The tables displayed information such as author names, countries, study designs, settings, participant samples, MNC measurement methods (see Table 2), key elements of MNC, perceived reasons for MNC, factors linked to MNC, and additional results (refer to Table 3). The tables in this paper were designed specifically to address the review questions.

### 2.5. Collating and Summarizing Data

This review employed the Synthesis Without Meta-analysis (SWiM) and PRISMA reporting guidelines to increase transparency, rigor, and reproducibility of the current narrative synthesis process [20]. Both SWiM and PRISMA are suitable for non-meta-analysis situations [20]. The data and synthesis included reading line-by-line and coding the text with letters (for MNC), or roman numbers (for reason for MNC and factors associated with MNC) to generate descriptive and analytical themes as recommended by Thomas and Harden’s three-step method for thematic synthesis [21], and this helped to achieve thematic synthesis [22].

The coding process helped to identify themes within and across studies that allowed for the characterization of MNC, reasons for the MNC, and associated factors. The first author conducted the initial coding and thematic synthesis by thoroughly examining and revisiting the findings from each study. The second and third authors independently checked and confirmed the original coding and thematic analysis. If any disagreements or fresh ideas came up, the reviewers gathered for a meeting to talk through their observations and work towards agreement. Consultations were conducted with stakeholders such as nurses working in inpatient oncology settings in Uganda, Oman, India, and the USA (countries where the authors have contacts and prior clinical practice and experience), to enhance external validity of the themes and subthemes. The themes and subthemes related to common MNC, reasons for MNC, and factors associated with MNC in inpatient oncology units were generated from the data summarized in Table 3 and these are presented in Table 4, Table 5 and Table 6.

**Table 2:** Summary of the setting, designs, participants, and measures of missed care used by the included studies.

Authors	Country	Study Design	Setting	Participants	MNC Measure
Albelbeisi et al. [23]	Palestine	Descriptive cross-sectional	Hospital-pediatric oncology wards	52 Nurses	MNC questionnaire developed by authors (α = not reported)
Dehgha-Nayeri et al. [24]	Iran	Inductive qualitative content analysis	Oncology units of hospitals	20 Nurse managers	Face-to-face interviews and focus group discussions using the interview guide
Friese et al. [25]	USA	Secondary analysis of survey data	Medical-surgical Units of 9 Hospitals in Midwestern states.	352 Nurses	MISSCARE survey (Test–retest coefficient = 0.87)
Jankowska-Polańska et al. [26]	Poland	Descriptive cross-sectional	Department of Pediatric Oncology, Hematology, and Bone Marrow Transplant in a university hospital	95 Nurses	BERNCA-R questionnaire (α = not reported)
Paiva et al. [27]	Portugal	Qualitative Descriptive	Inpatient units of an Oncology institution	10 Nurses	Semi-structured interview guide
Paiva et al. [28]	Portugal	Qualitative Descriptive	Inpatient units of an Oncology institution	10 Nurses	Semi-structured interview guide
Paiva et al. [29]	Portugal	Descriptive cross-sectional	Hospitals’ inpatient units exclusive for adult cancer patients	298 Nurses	MISSCARE survey Portuguese version (Overall scale α = 0.86)
Pan & Lin [30]	Taiwan	Descriptive cross-sectional	Private specialty cancer hospital (oncology wards)	111 Nurses	MISSCARE survey (Overall scale α = 0.90)
Papastavrou et al. [31]	Cyprus	Descriptive cross-sectional	Oncology-hematology units	157 Nurses	MISSCARE survey (Part A α = 0.957, part B α = 0.936)
Piotrowska et al. [32]	Poland	Descriptive cross-sectional	The oncology department of the hospital	100 Nurses	BERNCA-R questionnaire (α = not reported)
Rabin et al. [33]	Brazil	Descriptive cross-sectional	Inpatient oncology units of a private hospital	83 Nurses	MISSCARE survey (α = 0.927)
Shamsi et al. [34]	Iran	Descriptive cross-sectional	Oncology wards of multiple hospitals	93 Nurses	MISSCARE survey Persian version (α = not reported)
Villamin et al. [35]	USA	Descriptive, design	Six units of magnet-designated Comprehensive Cancer Centers	286 Nurses	MISSCARE survey (α = not reported)
Vryonides et al. [36]	Cyprus	Descriptive cross-sectional	Oncology-hematology units	157 Nurses	MISSCARE survey (Overall scale α = 0.90)
Ying et al. [37]	China	Multi Center Descriptive cross-sectional	Oncology hospitals in six provinces	446 Neuro-Oncology Nurses	Oncology Missed Nursing Care Self-Rating Scale (Overall scale α = 0.95)

Abbreviations: MNC, missed nursing care; BERNCA-R questionnaire, Basel Extent of Rationing of Nursing Care-Revised Questionnaire.

**Table 3:** Summary of the findings about the most frequently missed nursing care and the reasons for missed nursing care.

Authors	Country	Most Frequently Missed Care Element	Most Perceived Reasons for Missed Care	Factors Associated with Missed Nursing Care and Other Findings
Albelbeisi et al. [23]	Palestine	Oral hygiene Treatments and related procedures Clean the patient’s room and equipment Patient positioning and turning Transporting the patient within the hospital Performing morning nursing care Observe and monitor patients’ dietary intake Arrange discharge referrals and transportation Prepare patients and family for discharge Develop and update nursing care documentation Educating patients and family Respond to patient calls directly	Lack of adequate numbers of nurses Lack of opportunities for nurses to participate in policy decisions Lack of an active quality assurance program Management does not respond to employee concerns. Lack of a preceptor program for new nurses Lack of staff development, continuing education, and career development opportunities Lack of support staff and services to allow nurses to spend time with patients Lack of clear policies and procedures for nursing care	39% of nursing care is missed on oncology wards
Dehgha-Nayeri et al. [24]	Iran	Not stated	Staff shortages Heavy workload Performing repetitive, time-consuming tasks Substituting experienced nurses with unqualified relief nurses to compensate for staff shortages Having many newly qualified nurses Inappropriate delegation to nonspecialized staff (nurses’ assistants) Inadequate documentation and time-consuming documentation systems Shortage and nonfunctioning materials and equipment Delayed access to prescribed medications Perfunctory care Patient lack of health literacy Patient refusal of nurses of a different sex due to cultural restrictions Presence of visitors	Nurse managers defined MNC as arbitrary elimination of care, extended delays in healthcare service provision, or ineffective compensatory measures. Inefficient managers and lack of effective supervision Nurses’ lack of caring attitude Nurses’ lack of professional commitment Lack of teamwork Strained relationships between managers and nurses Cover-up by managers due to fear of being held accountable
Friese et al. [25]	USA	Ambulation (37%) Care conferences (25.3%) Mouth care (23%) Medication in less than 30 min (20.2%) Intake/output (17.1%) Feeding patients (15.6%) Turning every 2 h (13.9%) Documentation (14.9%) Responding to call light in >5 min (14.2%) Patient teaching (12.5%)	Not stated	A one-patient increase in the assignment was associated with a 2.1% increase in total MNC (*p* < 0.05). Perceived staffing adequacy was associated with MNC (*p* < 0.05).
Jankowska-Polańska et al. [26]	Poland	Perform adequate hand hygiene Not studying the information about the patient’s condition and care plan at the beginning of the shift Offering emotional or psychological support to a patient Having necessary conversations with a patient or their family Monitoring the patient as prescribed by the physician Administer a prescribed medication and/or infusion at the recommended time Assess the needs of newly admitted patients Document and evaluate the nursing care performed for a patient	Not stated	Greater fatigue levels among nurses were associated with higher levels of care rationing Care rationing was higher among nurses working 12-h shifts compared to those working 8-h shifts
Paiva et al. [27]	Portugal	Communication and discussion of confidential and complex topics Emotional support Feeding Oral hygiene tasks Oral hydration Adequate and clear documentation of care provided	Negligence or devaluation Inadequate training among assistants to whom tasks are delegated Ineffective delegation Willful misconduct or sloppiness Beliefs about the benefits of a type of care according to the patient’s clinical status Scarcity of human resources Workload and lack of time to meet patient demands Complexity of care provided to patients and their families Nurses’ physical and emotional exhaustion Lack of motivation and recognition for work done Lack of adequate skills in using computers and other technologies Structural conditions, such as the location of bathrooms and multiple patients in one room Insufficient material resources, such as adaptive equipment for hygiene care and lifting Organizational culture of a lack of acceptance of innovative solutions Communication failure among healthcare professionals Lack of teamwork Reprimands for nurses who make mistakes	Not stated
Paiva et al. [28]	Portugal	Communicating with the patient/family Educating the patient/family Keeping a record of nursing care provided/documentation. Updating of the care plans Oral hygiene Oral hydration Body hygiene care Positioning and repositioning of the patient Lifting the patient Assisting with ambulation Feeding Monitoring vital signs Monitoring capillary blood glucose Medication identification and administration within 30 min after prescription	Lack of record keeping Lack of time to reinforce teaching Complex digital platforms used for documentation. Existence of technologies such as viscoelastic mattresses that nurses do not know how to use. Lack of recognition of the value of some procedures by superiors, e.g., ambulation Workload Fear of the results of some assessments because they can trigger interventions and more work. Lack of knowledge about handling some medical devices	Nurses defined MNC as a part or whole of planned nursing care that is not performed Nurses believed that MNC can potentially worsen the patient’s condition Nurses believed that MNC can harm the family Nurses believed that MNC affects the nurse’s professional conscience Nurses believed that MNC leads to negative opinions about nursing care and the profession MNC compromises the transition from hospital to home due to failure to empower family caregivers MNC leads to suboptimal preparation of the family caregiver for home care
Paiva et al. [29]	Portugal	Ambulation three times per day or as ordered. Attend interdisciplinary care conferences whenever held	Unexpected rise in patient volume and/or acuity Inadequate number of staff Urgent patient situations Inadequate assistance and/or clerical personnel Heavy admission and discharge activity Unbalanced patient assignments Tension or communication breakdown with the medical staff	Nurses who worked 20–50 overtime hours reported more reasons for MNC. Nurses who worked 35 h a week reported fewer reasons for MNC. Personality traits of openness to experience, conscientiousness, and extraversion, when present, act as protective factors against MNC in the dimensions of instrumental care, patient assessment and documentation, and patient empowerment Older nurses missed less instrumental care Nurses’ satisfaction with their current position was protective against MNC. Intuitive style of decision making was associated with a higher incidence of MNC. A more analytical decision making process was a risk factor for MNC.
Pan & Lin [30]	Taiwan	Implementation of STAT orders for medication Assess the effectiveness of medications after they are administered. Handwashing at suitable times Completion of nursing records according to schedule Medication administered within 30 min of the scheduled time	Unexpected rise in inpatient volume Acuity of patients on the unit Urgent patient situations, e.g., condition worsening Insufficient number of nursing staff Heavy admission and discharge activity Main caregiver (family member or family caregiver) absent or unavailable	MNC was significantly associated with the nurses’ intention to resign and communication status Overall basic nursing-related care procedures were significantly associated with communication climate, horizontal and diagonal communication, informal communication, and organizational communication satisfaction. MNC was associated with human resource factors such as nurses’ level/seniority at work, experience, Unit manpower sufficiency, job title, and intention to leave
Papastavrou et al. [31]	Cyprus	Not stated	Inadequate number of staff Urgent patient situations Unexpected rise in patient volume/unit acuity Heavy admission and discharge activity Tension or communication breakdowns within the nursing team. Inadequate number of assistive and/or clerical staff	There was a significant positive relationship between job satisfaction and MNC No significant relationship between gender, age, work experience, intention to leave, and MNC
Piotrowska et al. [32]	Poland	Activation and rehabilitation interventions Administering prescribed medications or infusions at the right time Reviewing individual patient situations and care plans at the start of the shift Assessing the needs of newly admitted patients Preparing the patient and family for discharge	Not stated	Higher job satisfaction was associated with more frequent rationing of nursing care Greater emotional exhaustion, a stronger feeling of lack of personal accomplishment, and higher professional burnout were associated with less rationing of care
Rabin et al. [33]	Brazil	Assisting with toileting needs within 5 min of request Ambulation 3 times per day or as ordered Turning the patient every two hours Administering medications within 30 min before or after the scheduled time	Tension or communication breakdowns within the nursing team Caregiver responsible for the patient off the unit or unavailable Lack of backup support from team members Other professionals did not provide the care needed (e.g., physiotherapists did not ambulate patients) Supplies or equipment not working well when needed Heavy admission and discharge activity	Not stated
Shamsi et al. [34]	Iran	Participating in an interprofessional patient care conference Supervise food preparation for patients who can eat on their own Monitor feeding before food is cold Cooperation and supervision of the patient going to the toilet in the first 15 min of the request Emotional support for the patient and family	Unexpected increase in the number of patients or crowded wards Large volume of activities related to patient admission and discharge Lack of nursing staff Urgent patient situations, e.g., worsening of patients’ condition A large amount of information to be documented Engaging the nurse with other actions, such as secretarial duties Lack of support staff, e.g., secretary, assistants, and patient transporters Lack of support from team members The care mentioned is not related to the duties of the nurse	Not stated
Villamin et al. [35]	USA	Turning the patient every two hours Ambulation three times per day or as ordered Attend interdisciplinary care conferences whenever held Patient bathing/skin care Mouth care or oral care	Not stated	Surgical oncology units reported higher MNC scores Primary team nursing did not change the rate of MNC Rates of CAUTI were 0.8, 0.6, and 1.9 per 1000 catheter days on the medical, surgical, and hematology oncology units, respectively Rates of CLABSI were 0.6, 0.0, and 1.5 per 1000-line days on the medical, surgical, and hematology oncology units, respectively Rates of HAPU of ≥stage 2 were 0.0, 0.0, and 0.71 per 1000 patient days on the medical, surgical, and hematology oncology units, respectively Falls with injury rate was 0.57, 0.39, and 0.57 per 1000 patient-days on medical, surgical, and hematology oncology units, respectively
Vryonides et al. [36]	Cyprus	Attend interdisciplinary care conferences Turning the patient every 2 h Mouth care Patient teaching about illness, tests, and diagnostic studies Emotional support for patients and the family Ambulation three times per day or as ordered Patient discharge planning and teaching Feeding patients when the food is warm Assist with toileting needs within 5 min of request Medication administered within 30 min before or after the scheduled time Full documentation of all necessary data	Not stated	Nurses who perceive an ethical climate in the unit (benevolent, utilitarian ideals, compliance and respect for ethical principles, rules, laws, standards, and codes of conduct) reported fewer care omissions in their unit MNC was positively related to instrumental and independent ethical climates MNC was negatively related to the caring, rules, and the law and code of ethical climate
Ying et al. [37]	China	Incidence of MNC was 36.4% Most MNC was related to nursing assessment, care planning, and primary care Least risk for MNC was in the category of nursing interventions 20.6% of nurses were designated as having a severe risk for MNC profile 51.3% of nurses were designated as having a medium risk for MNC profile 28.1% of nurses were designated as having a slow risk for MNC profile	Not stated	Ages of 18–35 were associated with a low risk MNC profile (*p* < 0.001) Nurses with a technical secondary school or junior college education were more likely to be in the middle risk MNC profile (*p* = 0.011) Nurses satisfied with their position were more likely to be in the mediums risk MNC profile (*p* = 0.042) A positive work environment was associated with both low risk (*p* < 0.001) and middle risk (*p* < 0.001) profile. High mental self-confidence and mental toughness were associated with a medium risk profile (*p* = 0.006)

Abbreviations: MNC, missed nursing care.

**Table 4:** Sub-themes Under Commonly Missed Nursing Care in Inpatient Oncology Units.

Sub-Themes	Source/Citation
Medication administration	[23,25,26,28,30,32,33,36]
Proper documentation of nursing care	[23,25,26,27,30,36,37]
Assisting the patients with ambulation	[23,25,28,29,33,35,36]
Feeding or oral hydration	[23,25,27,28,34,36]
Oral hygiene	[23,25,27,28,35,36]
Positioning or turning of the patient	[23,25,28,33,35,36]
Proper patient assessment	[23,25,26,27,28,36,37]
Communication with patients and family	[23,26,27,28]
Attend multidisciplinary rounds & patient care conferences	[25,29,34,35,36]
Emotional and psychological support	[26,27,34,36]
Patient and family education	[23,25,28,36]
Proper discharge process	[23,32,36]
Toileting needs	[33,34,36]
Body hygiene	[23,28,35]
Hand hygiene	[26,30]
Monitoring vital signs	[28]
Laboratory testing	[28]
Patient supervision and monitoring	[26]
Activation of ordered or planned referrals	[32]
Clean the patient room and the patient care environment	[23]
Intake and output	[25]

**Table 5:** Sub-themes Under Reasons for Missed Nursing Care in Inpatient Oncology Units.

Sub-Themes	Source/Citation
Workload	[24,27,28,29,30,31,33,34]
Staff nurse shortage	[23,24,27,29,30,31,33,34]
Inadequate number and training of support staff	[23,27,28,29,31,33,34]
Unexpected rise in patient load	[24,27,29,30,31,33,34]
Urgent and emergent patient conditions	[27,29,30,31,34]
Nurses’ lack of skills related to technology	[24,27,28,34]
Poor communication among health professionals	[27,29,31,33]
Lack of teamwork	[27,31,33,34]
Presence of visitors or absence of family caregiver	[24,30,33]
Inappropriate delegation	[24,27,33]
Lack of supplies and equipment	[24,27,33]
Patient health illiteracy	[24,27,28]
Lack of organizational support for innovation	[27,33,34]
Lack of patient safety culture	[23,24,29]
Poor recordkeeping and documentation	[24,28,34]
Lack of motivation and recognition	[23,26,28]
Nurses’ beliefs and attitudes	[27,28]
Negligence	[27,28]
Inexperienced nurses	[23,24]
Delays due to inaction by other healthcare providers	[24,30]
Complexity of care	[27]
Physical and emotional exhaustion of the nurse	[27]
Structural limitations of the unit/layout	[27]
Lack of continuing education	[23]
Patient culture and beliefs	[24]

**Table 6:** Sub-themes Under Factors Associated with Missed Nursing Care in Inpatient Oncology Units.

Sub-Themes	Source/Citation
Job satisfaction	[29,31,32,37]
Unexpected increase in patient load	[25,26]
Perceived staff adequacy	[25,27]
Age of the nurse	[29,37]
Nurses’ formal education	[25,37]
Ethical climate in the unit	[36,37]
Emotional exhaustion and burnout	[32,37]
Lack of teamwork	[24]
Poor relationship with the managers	[24]
Lack of professionalism- nurse	[24]
Fatigue	[26]
Acuity of patient condition	[28]
Working overtime	[29]
Personality traits	[29]
Inefficient managers	[24]
Intuitive and analytical style of decision making	[29]
Intention to resign	[30]
Communication climate	[30]
Nurses experience	[30]

## 3. Results

Figure 1 displays the results of the search strategy and screening process. Of the 330 articles found in the search databases, fifteen (15) were selected for inclusion in the review. The results presented in Table 2 show that three studies used qualitative methods [24,27,28] and the other twelve used quantitative descriptive cross-sectional methods [23,25,26,29,30,31,32,33,34,35,36,37]. The lack of mixed-method studies and interventional studies indicates a gap that needs to be addressed. Many of the studies were conducted in European countries such as Portugal [27,28,29], Poland [26,32], and Cyprus [31,36]. The remaining studies were from Iran [24,34], Palestine [23], Taiwan [30], the USA [25,35], Brazil [33], and China [37]. There were no studies from countries in Africa and Australia that specifically reported on MNC in inpatient oncology units or settings. There were also no randomized controlled trials or intervention studies.

The most used scale to measure MNC or reasons for MNC by the quantitative studies was the MISSCARE survey [25,29,30,31,33,34,35,36], and this was dependable with Cronbach’s alpha ranging from 0.86 to 0.96 [25,29,30,31,33,36]. The other quantitative studies used the BERNCA-R questionnaire [26,32], the oncology missed nursing care scale [37], or other unstandardized scales [23]. The predominant use of the MISSCARE survey indicates its relevance and validity in accurately measuring the phenomenon of missed nursing care in various countries. The were only two studies that focused on pediatric oncology care settings [23,26], and this indicates another gap in knowledge and research on MNC. The analysis and synthesis of the data led to three overall themes, and these include common missed nursing care, reasons for missed nursing care, and factors associated with missed nursing care.

### 3.1. Common Missed Nursing Care

Multiple studies on the MNC identified twenty sub-themes within the broader theme of common MNC in inpatient oncology units. In this instance, the sub-themes matched the types of MNC and can be found in Table 4. The most commonly reported MNC by most studies were aspects related to basic patient care, such as medication administration [23,25,26,28,30,32,33,36], documentation of nursing care [23,25,26,27,30,36,37], assisting patients with ambulation [23,25,28,29,33,35,36], feeding or oral hydration [23,25,27,28,34,36], oral hygiene [23,25,27,28,35,36], patient turning and positioning [23,25,28,33,35,36], and others. Failure to perform the above aspects of basic nursing care could increase the patient’s risk for nosocomial infections, pressure ulcers, deep vein thrombosis, and other complications. The highlighted MNC also has potentially unsafe practices that need urgent attention to ensure high-quality nursing care in inpatient oncology units.

### 3.2. Reasons for Missed Nursing Care

Under the main theme of reasons for MNC, there are twenty sub-themes (each representing a reason for MNC as reported by the nurses). These themes represent the rationale directly reported by nurses as the basis for the MNC. Table 5 summarizes the reasons for MNC, and it is evident that issues involving human resources and workload are the most frequent. The most commonly reported reasons for MNC were workload [24,27,28,29,30,31,33,34], staff nurse shortage [23,24,27,29,30,33,34], inadequate number of trained support staff [23,27,28,29,31,33,34], unexpected rise in patient load [24,27,29,30,31,33,34], urgent and emergent patient conditions [27,29,30,31,34], nurses’ lack of skills related to technology [24,27,28,34], poor communication [27,29,31,33] and teamwork [27,31,33,34].

The reasons highlighted above as the basis for MNC show a situation where standards, policies, or guidelines related to nurse-to-patient ratio, communication, delegation, management of surges in patient load, and others are either not followed or do not exist. This could be due to a failure to adopt evidence-based practice in inpatient oncology settings. There are evidence-based tools and standard approaches for communication and handover report, teamwork and team building, and managing upsurges in patient volumes or during crises that could be adopted for use in inpatient oncology settings. Although such standards and tools may have been developed in non-oncology settings, they can be adapted, refined, and applied to reduce MNC and enhance the quality of care.

The reasons related to physical and emotional exhaustion of the nurse and complexity of the care provided in inpatient oncology settings were not commonly reported. This was surprising in view of reports showing that job satisfaction [29,31,32,37], burnout [32,37], and fatigue [26] are significantly associated with MNC. It is important to note that the reasons for MNC also highlight deficiencies in the nurse managers that lead nurses in the inpatient oncology units. The deficiencies in management and leadership provide a fertile ground for work environments with no teamwork, poor supervision, poor reporting of MNC, poor communication, high work-related stress, and low utilization of evidence-based practice in clinical practice. Therefore, nurse managers of units that provide complex nursing care, such as inpatient oncology units, need to acquire specialized training and skills to provide effective leadership to sustain a work environment that ensures high patient safety, quality nursing care, and professional well-being of the nurses.

### 3.3. Factors Associated with Missed Nursing Care

The findings of studies that reported on MNC and the factors associated with MNC led to the broad theme of factors associated with MNC (see Table 6). The factors associated with MNC include aspects that were not directly reported by the nurses (as reasons for missing the care) but were generated during statistical analyses and found to be significantly associated with MNC. In some instances, the factors associated with MNC are closely similar to the rationale directly stated by nurses for the MNC. The major factors associated with MNC (reported by at least two studies) were related to nurses and their workplace environment. The common factors were job satisfaction [29,31,32,37], unexpected increase in patient load [25,26], perceived staff adequacy [25,27], age of the nurse [29,37], ethical climate on the unit [36,37], and emotional exhaustion and burnout [32,37].

The factors commonly associated with MNC highlight the importance of maintaining a conducive work environment, nursing human resource capacity, and nurses’ professional quality of life. A work environment with adequate emphasis on the above three attributes is likely to adequately manage factors such as job satisfaction [29,31,32,37], ethical climate [36,37], intention to resign [30], fatigue [26], burnout [32,37], and others presented in Table 6. Work environments such as inpatient oncology units need to implement deliberate interventions to enhance or maintain nurses’ professional quality of life. One study that gathered information from nurse managers working in inpatient oncology units also identified obstacles to reporting MNC. The main barriers to reporting MNC included inadequate staff supervision, the nurses’ attitudes and professionalism, the relationship between nurses and their managers, and the lack of patient safety culture [24].

## 4. Discussion

This is the first review to synthesize evidence on MNC in inpatient oncology settings, and it provides new knowledge that is critical to oncology nursing care and practice. The findings of the review characterize the commonly missed nursing care that is key to patient safety, safe nursing care, the healing process, and prevention of complications in inpatients with cancer and cancer survivors admitted to oncology units. Such aspects include missing medication administration [23,25,26,28,30,32,33,36], poorly documented or undocumented nursing care leading to a lack of continuity of care [23,25,26,27,30,36,37], failure to assist the patient with activities of daily living such as ambulation, feeding, oral hydration, oral hygiene, positioning, or turning of the patient, and others. The above instances of MNC are essential to the patients’ recovery and nursing care, and gaps in the above domains are likely to increase the length of stay in the hospital, reduce patient satisfaction, increase risk of infections, mortality, and other undesirable outcomes.

The other findings of the review show that emotional and psychological support [26,27,34,36], patient assessment [23,25,26,27,28,36,37], communication with patient and family members [23,26,27,28], and proper discharge process [23,32,36] are frequently missed in inpatient oncology care settings. Cancer patients and survivors often need psychological support and effective communication because they struggle with understanding their illness and treatment options, do not know what to expect, experience severe emotional impact of the illness, lack control over their lives, and suffer spiritual, sexual, and financial problems [38]. These problems become even more important during cancer treatment and the survivorship period. Thus, our findings highlight emotional and psychological support as a key area of concern that needs to be addressed by efforts to improve the quality of oncology nursing care across the cancer disease trajectory.

MNC such as proper patient assessment [23,25,26,27,28,36,37], proper discharge process [23,32,36], patient and family education [23,25,28,36], communication with patients and family [23,26,27,28], and activation of ordered or planned referral [32] can grossly impact continuity care, patient’s satisfaction with nursing care [39], and readiness for selfcare while at home. It is correct that MNC leads to suboptimal preparation of the patient and family caregiver for home care [28]. And it is logical to conclude that MNC can lead to safety issues while the patient is in the hospital and when they return home after discharge, because MNC compromises the transition from hospital to home due to failure to empower the patient and family caregivers.

The results also highlight that indirect nursing care is missed a lot, and this includes aspects such as proper documentation of nursing care [23,25,26,27,30,36,37] and attending multidisciplinary rounds and patient care conferences [25,29,34,35,36]. These processes are essential since this is when treatment plans, priorities, and vital information about patient needs and future care are discussed. Nurses’ failure to attend to these is likely to increase the chances of the patient having unmet needs.

In this review, we also found that the most reported reasons for MNC were related to nursing human resources, workload, communication, and other skills of the nurse. These are essential areas that need to be targeted with interventions to improve the quality of nursing care provided to hospitalized cancer patients and survivors. One of the needed interventions that can help address the shortage of nursing human resources is the opening of programs that train oncology nurses in countries where these are lacking. Nurses with specialized skills and competencies in oncology may have a lower propensity for MNC and may be the substrate needed to enhance the quality of oncology nursing care. The MNC and its impact on the quality of nursing care requires a capacity-building response because oncology nurses are at the heart of tackling the increasing global burden of cancer [40]. The contribution of oncology nurses is unique because of the complexity and the diversity of care roles and responsibilities they assume in cancer care [40].

The diversity and frequency of MNC established by this scoping review also highlights the increasing need for personalized nursing care in oncology nursing. This implies that inpatient nursing care models that emphasize personalized nursing care are likely to have less MNC. Moreover, individualized nursing care is associated with a favorable perception of high-quality oncology nursing care and health outcomes [41]. The frequency and diversity of MNC also indicates the need to establish MNC as a quality indicator in oncology nursing and cancer care services. And such a quality indicator needs to be regularly monitored and reported the same way pressure injuries and falls are monitored. Consistent tracking and monitoring of MNC could reduce their incidence and subsequently enhance oncology nursing care outcomes.

The results of this review also reinforce the view that many interconnected elements—such as continuous education, nursing expertise, staffing levels, teamwork across disciplines, and workplace conditions—influence the quality of oncology nursing care [42]. And quality oncology nursing care can also increase the rate of cancer survivors. Thus, addressing MNC and the associated factors using tailored and comprehensive interventions is essential for optimizing patient outcomes and oncology nursing care across the world.

A comprehensive approach to curtailing MNC and enhancing the quality of oncology nursing care should include policies, clinical practice guidelines, regular monitoring, nurses’ education, and resource optimization. As a starting point, healthcare organizations should prioritize strategies that support nurses in providing optimal care, such as continuous education and training focusing on care across the cancer disease trajectory and fostering a supportive work environment that promotes teamwork and staff well-being [42]. Recognizing the significance of effective interdisciplinary collaboration and the availability of necessary resources is crucial in enhancing the overall quality of care provided to oncology patients [42].

The most significant implication of MNC is its impact on patient safety. Globally, patient safety culture research holds paramount importance for the healthcare sector, and it is a critical concern [43]. Patient safety is recognized by the Institute of Medicine (IOM) as a cornerstone within healthcare systems, without which improvements in the overall quality of care are impossible [44]. IOM entrenches the need to establish a safety-oriented culture within healthcare organizations, intending to enhance patient safety and overall quality of care. Reduction in MNC ensures patients’ safety. Thus, prioritizing MNC not only enhances patient safety and reduces errors but also promotes the well-being of healthcare workers and enhances the overall quality of care within healthcare organizations [44]. Nurses working in inpatient oncology care settings need to be familiar with initiatives such as the Global Patient Safety Action Plan adopted by the World Health Organization (WHO) to prevent avoidable harm, promote patient safety across practice domains, and help countries develop national action plans [45]. Such efforts are crucial to reducing MNC because they emphasize strengthening policies and strategies rooted in scientific evidence and patient feedback, as well as building national policies consistent with the global goals of a culture of patient safety. The overall goal is to reduce risks and preventable harm from aspects such as MNC that can result in negative outcomes for patients and nurses. According to the WHO, investing in aspects such as safety and reduction of MNC is crucial for achieving positive organizational outcomes [46].

Furthermore, as the global cancer burden grows, there is a need for more research—including interventional and mixed method studies from underrepresented regions to better understand MNC. This review presents the first synthesis of findings from studies about MNC in inpatient oncology settings, and there were only fifteen accessible studies from across the world about MNC in oncology care settings. The review included only two studies with a focus on pediatric oncology and did not find studies or articles based on studies in inpatient oncology settings in Africa, Australia, Southeast Asia, and the Caribbean. The fact that most of the studies were from Western countries and published in one language (English) skews the knowledge we have about MNC in inpatient oncology settings with a Western perspective. This is a gap in knowledge that undermines our understanding of the regional and true global status of MNC in oncology settings. There is still a need for studies about MNC in pediatric and adult inpatient oncology care settings from other parts of the world to enable us to develop a more comprehensive picture of the MNC, the reason behind MNC, and potential solutions that are applicable in the diverse cancer care systems across the world.

### 4.1. Relevance to Clinical Practice

The common instances of MNC identified, along with their underlying reasons, highlight the risks and unsafe care that patients encounter in inpatient oncology units. Thus, the knowledge generated by this review delineates aspects of oncology nursing care that clinicians, researchers, policy makers, and hospital administrators need to focus on while seeking to enhance nursing care and patient outcomes in inpatient oncology units. The MNC such as failure to perform medication administration on time, feeding and oral hydration, oral hygiene, assisting patients with ambulation, positioning or turning of the patient, emotional and psychological support, communication with patient and family members, proper discharge processes, documentation of nursing care, and others, subsequently reduce the quality nursing care, patient safety, and can reduce the chances of surviving cancer.

The problem of workload, staff shortage, and lack of specific skills by nurses highlights the role of nurse educators and nurse training institutions across the world. There is a critical shortage of oncology nursing human resources to provide the complex and specialized nursing care required by cancer patients. We believe that one approach to addressing the issue of MNC in oncology is to increase the number of specialized oncology nurses with the skills and competencies needed to meet the needs of this patient population. To achieve this boast in nursing human resources, governments, policy makers, nurse educators, and training institutions need to work synergistically to ensure a coordinated approach to developing and utilizing these resources. We recommend more programs to train nurses in the oncology nursing specialty, especially in low to middle-income countries where the future burden of cancer will be the highest.

Factors such as workload, unethical work environment, lack of teamwork, and poor communication indicate a need for nurse leaders and managers in inpatient oncology units with skills and competencies to sustain a conducive work environment and healthy workforce. Moreover, many of the MNC instances and reasons for MNC can be addressed by increased utilization of evidence-based practice and standardized tools. However, competent and skilled nurse leaders and role models are needed to increase the penetration of evidence-based practice in the clinical setting and to mentor nurses in the utilization of evidence-based practices.

Increasing instances of MNC have severe implications for hospitals and healthcare systems since they can fuel increased healthcare costs through increased nosocomial infections, hospital re-admissions, and increased length of hospital stay. Thus, healthcare organizations and healthcare systems that desire to provide quality oncology nursing care must institute systems that regularly audit MNC and act to reduce such incidences. It is important to note that as the population’s health literacy increases and patients become more aware of the expected quality of nursing care, in the future, MNC will lead to increased lawsuits or legal actions against nurses and their employers. Increased legal action against healthcare organizations will alarm governments, regulatory authorities, and insurance companies. An increase in legal action against nurses will also fuel more nursing shortages and increased healthcare costs. A key part of the defense against unsafe practice, morbidity, mortality, and legal action resulting from MNC is evidence-based practice. We call upon all inpatient oncology care settings to integrate evidence-based practice in all aspects of nursing care as a preventative and quality assurance strategy.

We recommend the implementation of systems to regularly monitor MNC and reasons for MNC in inpatient oncology units as a way of supporting continuous quality improvement processes and enhancing chances of surviving cancer. We believe MNC is a key indicator of the quality of nursing care and should be a benchmark of the quality of oncology nursing care. Moreover, such a benchmark is achievable by implementing interventions to reduce the incidence of MNC, such as practicable nursing workload, adequate nurse staffing and qualified support staff, policies and procedures to follow during an unexpected rise in patient load, updated protocols, guidelines, and standards for communication among healthcare professionals, skilling of nurses is aspect related to technology, and others. Standardized tools like the MISSCARE survey, BERNCA-R, and the oncology missed nursing care self-rating scale are available for evaluating conditions both prior to and after interventions.

### 4.2. Strengths and Limitations of the Study

The review demonstrates strengths that include the comprehensive systematic database search that was conducted, as well as the process by which the authors independently assessed eligibility and the extracted data. Secondly, the data were analyzed and discussed by the research team, enhancing the study’s credibility and intersubjectivity. Thirdly, we followed an acknowledged methodological framework used to conduct scoping reviews. The current review considered studies from across the world that focused on MNC in inpatient oncology settings. Consultation with stakeholders, which is an important part of a scoping review, were conducted with stakeholders (nurses working in inpatient oncology settings) in Uganda, Oman, India, and the USA (countries where the authors have contacts and prior clinical practice). But these are limited considering the diversity of healthcare systems in the world. To our knowledge, this is the first review to summarize evidence from primary studies focusing on MNC in inpatient oncology settings. Thus, the review helped to map the common MNC and reasons for MNC in inpatient oncology settings across the world.

As with every study, there are also limitations. The first of these is that we only included studies published in English, in the period ranging from 2013 to 2025. As such, some relevant papers could be missing if they were published in other languages or outside the stated period. Another limitation is access to gray literature. There could be some theses, papers, and studies published in forums for gray literature, which we did not have access to, and this may also have led to the exclusion of relevant literature. Moreover, the critical appraisal of individual sources of evidence is not seen as relevant to a scoping review and was deemed beyond the scope of this article. Therefore, the quality of the included studies was not assessed (so the risk of bias and the validity of the included studies are unknown). Another limitation is that many patients with cancer are taken care of in hospital units that are not specialized inpatient oncology care settings. Nurses working on such general units may have participated in studies focusing on MNC that were not included in the review. This may have led to the exclusion of relevant literature. We have provided a Supplementary File titled “search details”. However, in an ideal world, we could have the protocol published before the study to enhance further transparency.

## 5. Conclusions

The omission of essential nursing care in oncology settings is a significant issue that compromises patient safety, patient health outcomes, and the chances of surviving cancer. This scoping review found that the most common instances of MNC include basic patient care, emotional support, education, and documentation. The primary reasons for MNC were those related to human resources, staffing, workloads, communication, and the work environment. No intervention studies have examined MNC in inpatient oncology units, nor have any been conducted in these specific patient populations or care settings in regions like Africa, where the future cancer burdens are expected to be highest. Addressing these gaps requires systemic reforms, including research, guidelines, and policies to foster patient safety and a supportive work environment.

## Figures and Tables

**Table 1:** Search strategy.

Keywords Search terms (all databases) MeSH or Thesaurus terms	Missed care, Missed nursing care, task undone, unfinished care, rationed care, care left undone, delayed care, nurse, nurses, cancer nurses, oncology nurses, oncologic care, chemotherapy, radiotherapy * OR nursing AND neoplasm N1 (missed care OR rationed care OR care left undone) Missed care (CINAHL, Medline, PsycINFO, EMBASE, Scopus); missed nursing care (CINAHL); Nurse, Oncology nursing (CINAHL, Medline); Nursing care, Nurse, Oncology Nurse (PsycINFO, Scopus)

* represents a truncation or wildcard character to broaden search results.

## Data Availability

No new data were created.

## Supplementary Materials

The following supporting information can be downloaded at: https://www.mdpi.com/article/10.3390/nursrep15120413/s1.

## Public Involvement Statement

There was no public involvement in any aspect of this research.

## Guidelines and Standards Statement

This scoping review was prepared following both the SWiM and PRISMA guidelines [20], as well as the five-stage scoping review framework [18,19].
