# Supplementary material for: A Global Overview of Missed Nursing Care During Care of In-Patients with Cancer: A Scoping Review
Source: Nurs Rep. 2025 Nov 24;15(12):413. doi: 10.3390/nursrep15120413 (PMC12735627; doi:10.3390/nursrep15120413)
Supplement: Supplementary file 1 [file nursrep-15-00413-s001.zip › nursrep-3977604-supplementary.pdf]

Search Strategies for the Scoping Review – "Missed Nursing Care in Inpatient Oncology Units"

CINAHL

S1	TI (missed care N1 (task undone OR unfinished care OR rationed care)) OR AB missed care N1 (delayed care OR care left undone)) AND (Neoplasms OR Oncological Care OR Chemotherapy OR Radiotherapy)
S2	(MH "Missed Nursing Care") OR (MH "Rationed Care") AND (Neoplasms OR Oncological Care OR Chemotherapy OR Radiotherapy)
S3	S1 OR S2
S4	TI (nurs* OR nursing OR cancer nursing OR oncology nursing) OR AB (nurs* OR nursing OR cancer nursing OR oncology nursing)
S5	(MH "Nursing, Nurses, Nursing Practice, Nurse Navigator")
S6	S4 OR S5
S7	S3 AND S6

Key *– TI (title), N1 (near one), AB (abstract), MH (medical subject headings)*

Medline

S1	TI (missed care N1 (task undone OR unfinished care OR rationed care)) OR AB missed care N1 (delayed care OR care left undone)) AND (Neoplasms OR Oncological Care OR Chemotherapy OR Radiotherapy)
S2	(MH "Missed Nursing Care") OR (MH "Rationed Care") AND (Neoplasms OR Oncological Care OR Chemotherapy OR Radiotherapy)
S3	S1 OR S2
S4	TI (nurs* OR nursing OR cancer nursing OR oncology nursing) OR AB (nurs* OR nursing OR cancer nursing OR oncology nursing)
S5	(MH "Nursing, Nurses, Nursing Practice, Nurse Navigator")
S6	S4 OR S5
S7	S3 AND S6

Key *– TI (title), N1 (near one), AB (abstract), MH (medical subject headings)*

APA PsycINFO and ERIC

S1	TI (missed care N1 (task undone OR unfinished care OR rationed care)) OR AB missed care N1 (delayed care OR care left undone)) AND (Neoplasms OR Oncological Care OR Chemotherapy OR Radiotherapy)
S2	DE "Missed Nursing Care"
S3	S1 OR S2
S4	TI (nurs* OR nursing OR cancer nursing OR oncology nursing) OR AB (nurs* OR nursing OR cancer nursing OR oncology nursing)
S5	DE "Nurses"
S6	S4 OR S5
S7	S3 AND S6

Key *– TI (title), N1 (near one), AB (abstract), DE (phrase indexed keyword)*

Searches via other methods

Resource	Details	Number found
Trove	Searched conference papers and reports using keywords – *missed care* and n*ursing* (2013-2025)	0
ProQuest Dissertations	Searched using keywords *missed care* OR *rationed care OR unfinished care AND nursing*	1 (already published as a paper)
Google Scholar	Searched using keywords *missed care* OR *rationed care OR unfinished care AND nursing*	15 articles and 1 dissertation
